# Comprehensive Transcriptome Analysis Reveals the Role of lncRNA in Fatty Acid Metabolism in the Longissimus Thoracis Muscle of Tibetan Sheep at Different Ages

**DOI:** 10.3389/fnut.2022.847077

**Published:** 2022-03-14

**Authors:** Gaoliang Bao, Shaobin Li, Fangfang Zhao, Jiqing Wang, Xiu Liu, Jiang Hu, Bingang Shi, Yuliang Wen, Li Zhao, Yuzhu Luo

**Affiliations:** Gansu Key Laboratory of Herbivorous Animal Biotechnology, Faculty of Animal Science and Technology, Gansu Agricultural University, Lanzhou, China

**Keywords:** lncRNA, skeletal muscle development, fatty acid metabolism, Tibetan sheep, regulation of nutrient metabolism

## Abstract

Long noncoding RNA (lncRNA) plays an important regulatory role in mammalian adipogenesis and lipid metabolism. However, their function in the longissimus thoracis (LT) muscle of fatty acid metabolism of Tibetan sheep remains undefined. In this study, fatty acid and fat content in LT muscle of Tibetan sheep were determined, and RNA sequencing was performed to reveal the temporal regularity of lncRNA expression and the effect of lncRNA-miRNA-mRNA ceRNA regulatory network on lipid metabolism of LT muscle in Tibetan sheep at four growth stages (4-month-old, 4 m; 1.5-year-old, 1.5 y; 3.5-year-old, 3.5 y; 6-year-old, 6 y). The results indicated that the intramuscular fat (IMF) content was highest at 1.5 y. Moreover, the monounsaturated fatty acid (MUFA) content in 1.5 y of Tibetan sheep is significantly higher than those of the other groups (*P* < 0.05), and it was also rich in a variety of polyunsaturated fatty acids (PUFA). A total of 360 differentially expressed lncRNAs (DE lncRNAs) were identified from contiguous period transcriptome comparative groups of 4 m vs. 1.5 y, 1.5 y vs. 3.5 y, 3.5 y vs. 6 y, and 4 m vs. 6 y, respectively. Kyoto encyclopedia of genes and genomes (KEGG) enrichment analysis found that the target genes in lncRNA trans-mRNA were significantly related to the protein digestion, absorption, and fatty acid biosynthesis pathways (*P* < 0.05), which demonstrated that DE lncRNA *trans*-regulated the target genes, and further regulated the growth and development of the LT muscle and intramuscular fatty acid metabolism in Tibetan sheep. We further analyzed the role of the lncRNA-miRNA-mRNA regulatory network in the lipid metabolism of Tibetan sheep. Additionally, *GPD2, LIPE* (lipase E hormone-sensitive enzyme)*, TFDP2, CPT1A, ACACB, ADIPOQ*, and other mRNA related to fatty acid and lipid metabolism and the corresponding lncRNA-miRNA regulatory pairs were identified. The enrichment analysis of mRNA in the regulatory network found that the AMPK signaling pathway was the most significantly enriched (*P* = 0.0000112361). Comprehensive transcriptome analysis found that the *LIPE, ADIPOQ, ACACB*, and *CPT1A* that were regulated by lncRNA might change the formation of energy metabolism in Tibetan sheep muscle through the AMPK signaling pathway, and oxidized muscle fibers are transformed into glycolytic muscle fibers, reduced IMF content, and the fatty acid profile also changed.

## Introduction

Tibetan sheep is the dominant livestock resource in the Qinghai-Tibet Plateau, living at an altitude above 3,000 m on the Qinghai-Tibet Plateau with high altitude, low temperature, thinner oxygen, and strong UV radiation. Tibetan sheep meat is loved by consumers who like mutton for its unique flavor (1, 2). Skeletal muscle generation is an extremely complex and precise process, which is the result of the interaction between genetics and environment, including sequential steps of muscle stem cell proliferation, myoblast differentiation, and cell fusion to form multinucleated myotubes and so on (3). Mammalian skeletal muscle is a heterogeneous tissue, which is composed of various muscle fibers that exhibit different physiological and metabolic properties, such as glycolysis, oxidative metabolism, and contraction. Skeletal muscle is the major component of body mass accounting for approximately 50% of body mass in a mammal (4). Skeletal muscle growth and development directly influence muscle quality and meat production. Meanwhile, different muscle fiber types of skeletal muscle also have an influence on intramuscular fat (IMF), meat tenderness, water retention, juiciness, and fatty acid composition (5, 6). In addition, the IMF also determines the meat tenderness and fatty acid profiles in the skeletal muscle (7). Fatty acids are an important part of cells and are involved in the energy metabolism of human and animals as signaling molecules (8). Therefore, exploring the mechanism of skeletal muscle growth and development is very important to improve lipid and fatty acid metabolism in Tibetan sheep meat.

Previous studies demonstrated that 80% of the mammalian genome can be transcribed, and RNA is produced from many expressed genomic sites in the sense and antisense DNA strands (9). Additionally, long noncoding RNA (lncRNA) is a type of RNA transcript from the sense strand of DNA, over 200 nucleotides in length, no or weak protein-coding potential, low expression, with specificities of tissue and developmental stage, and low conservation among different species (10–12). It serves as a key functional molecule that mediates a variety of biological processes in organisms, such as histone modification, chromosome remodeling, gene expression regulation, miRNA precursor generation, animal development, cell differentiation, and so on, which is a hot topic of this research (13, 14). lncRNA is essential for the physiological regulation of cell proliferation and differentiation (15). A large number of studies have found that lncRNA has an important regulatory effect on fatty acid and lipid metabolism in human and animals (16, 17).

Long noncoding RNAs can regulate the expression of functional genes in a variety of ways. lncRNAs regulate the expression of neighboring functional genes through *cis*-mRNA and can also regulate distant target genes through *trans*-mRNA (18). In addition, lncRNAs could serve as miRNA sponges and thereby impair miRNA-mediated gene silencing (19). Jiang et al. (20) found that differentially expressed lncRNAs (DE lncRNAs) might play an important role in the adipose tissue of cattle at different age groups (20). Zhang et al. (3) found that lnc403 influenced the differentiation of skeletal muscle cells by regulating the expression of neighboring genes and interacting proteins and revealed that lnc403 might participate in the differentiation of bovine myoblasts (3). Yang et al. (21) also demonstrated that Gm16551 was a negative regulator of lncRNA related to SREBP1c activity and adipogenesis in mouse liver (21). Yue et al. (22) constructed a potential function of ceRNA network in bovine skeletal muscle, which indicated that lncRNA had functional specificity for bovine skeletal muscle development, and DE lncRNA was significantly enriched in the biological processes related to muscle development and the Wnt signaling pathway (22). Many studies have demonstrated several lncRNAs involved in mammalian adipogenesis and lipid metabolism. However, there are no studies on the lncRNA in Tibetan sheep muscle development, IMF, and fatty acid metabolism. A total of sixteen Tibetan sheep in four growth stages was selected in this study based on the differences in slaughter performance and meat quality, and the differences in lipid metabolism at different growth stages were analyzed. We further analyzed the function of lncRNA and the mechanism of the lncRNA-miRNA-mRNA ceRNA regulatory network in the longissimus thoracis (LT) muscle lipid metabolism of Tibetan sheep using an RNA sequencing approach. We analyzed the function and regulatory network of lncRNA in lipid metabolism during muscle growth and development, especially with the increase of age, and explored the role of lncRNA in the transformation of oxidized muscle fiber and glycolytic muscle fiber in Tibetan sheep and its influence on the difference of fatty acids, which would benefit to explore the molecular mechanism of Tibetan sheep muscle growth and development.

## Materials and Methods

### Animals and Muscle Sampling

Sixteen healthy female Tibetan sheep were randomly selected from the same sheep flock of Haiyan County, Qinghai Province, China (3,500 m above sea level), including 4-month-old, 4 m (*n* = 4); 1.5-year-old, 1.5 y (*n* = 4); 3.5-year-old, 3.5 y (*n* = 4); and 6-year-old, 6 y (*n* = 4). In addition, 4 m, 1.5 y, 3.5 y, and 6 y represent the lambs, the pubertal sheep, the adult sheep, and the old sheep, respectively. All sheep had the same nutrition and were raised under the same environmental conditions with natural light and free access to food and water. Four Tibetan sheep from each growth stage were weighed and immediately slaughtered humanely according to the Islamic practice (exsanguinated, peeled, and split down the midline according to standard operating procedures). The experiment was conducted in accordance with the guidelines of the National Institutes of Health Guide for the Care and Use of Laboratory Animals (NIH Publications No. 8023, revised 1978), and animal welfare and conditions were considered in the use of experimental animals. The carcasses were placed in a chilling room at 4°C before sampling for meat quality traits. LT muscle (from 12th thoracic vertebrae to 5th lumbar vertebrae) samples from the left half carcass were collected from the four different growth stages after slaughtering and immediately frozen in liquid nitrogen and stored at −80°C for RNA isolation.

### Meat Quality Measurements

#### IMF Content

The IMF content was measured using the Soxhlet extraction method with solvent (petroleum ether) and expressed as a weight percentage of wet muscle tissue (AOAC, 2007), with three replicates for each sample (23).

#### Fatty Acid Profile

Fatty acids were extracted from LT muscle samples of Tibetan sheep and methylated as described by Gao et al. (24). The fatty acid profiles were analyzed by gas chromatography (SRI Model 8610C, USA). The nitrogen flow rate was set to 1.2 ml/min, and the airflow rate was set to 450 ml/min. The column was operated isothermally at 140–240°C at 5°C/min and kept at 240°C for 15 min. The injection and detector temperatures were 260°C and 250°C, respectively. Hydrogen (40 ml/min) was used as the carrier gas. Fatty acids were qualitatively identified by comparing the retention times of 37 fatty acids (Supelco 37 FAME Mix 47885-U, USA) and quantified by comparison of the peak areas of the sample and the internal standard (C11:0). The results were reported as grams of fatty acid per 100 g of LT muscle samples. The nutritional properties of LT muscle samples of Tibetan sheep were evaluated by calculating the ratios of PUFA/SFA and n-6/n-3 fatty acids.

#### RNA Extraction, Strand-Specific Library Construction, and Sequencing

The total RNA was extracted using the Trizol Reagent Kit (Invitrogen, Carlsbad, CA, USA) according to the manufacturer's protocol. RNA quality was assessed on an Agilent 2100 Bioanalyzer (Agilent Technologies, Palo Alto, CA, USA) and checked using RNase-free agarose gel electrophoresis. After the total RNA was extracted, rRNAs were removed to retain mRNAs and ncRNAs. The enriched mRNAs and ncRNAs were fragmented into short fragments by using fragmentation buffer and reverse transcribed into cDNA with random primers. Second-strand cDNA was synthesized by DNA polymerase I, RNase H, dNTP (dUTP instead of dTTP), and buffer. Then, the cDNA fragments were purified using the QiaQuick PCR Extraction Kit (Qiagen, Venlo, the Netherlands), end-repaired, a base added, and ligated to Illumina sequencing adapters. Then, uracil-*N*-glycosylase (UNG) was used to digest the second-strand cDNA. The digested products were size selected by agarose gel electrophoresis, PCR amplified, and sequenced using Illumina Novaseq6000 (or other platforms) by Gene Denovo Biotechnology Co., Guangzhou, China.

#### Transcriptome Assemble

The reads containing adapters were filtered using fastp (version 0.18.0) (25), removing reads containing more than 10% of unknown nucleotides (N) and low-quality reads containing more than 50% of low quality (*Q*-value ≤ 20) bases, to get high-quality clean reads. Short read alignment tool Bowtie 2 (version 2.2.8) was used for mapping reads to the ribosome RNA (rRNA) database (26). The rRNA mapped reads were then removed. The remaining clean reads were mapped to the reference genome Oar_rambouillet_v1.0 using HISAT2 (version 2.1.0) (27). The reconstruction of transcripts was carried out using StringTie (version 1.3.4) (28, 29).

#### lncRNA Identification

The filter criteria of lncRNAs were as follows: (a) seven classes of transcripts (“i,” “j,” “x,” “u,” “c,” “e,” and “o”) were defined as a novel transcript; (b) the length of the transcript was longer than 200 bp, and the exon number more than 2 was identified a reliable novel gene; (c) transcripts that overlap protein-coding mRNAs were removed; (d) novel transcripts were then aligned to the Nr, Kyoto encyclopedia of genes and genomes (KEGG), and gene ontology (GO) database to obtain protein functional annotation and exclude known mRNA and other noncoding RNAs (such as rRNA, tRNA, snoRNA, and snRNA). Transcripts without coding potential, as predicted by CNCI (version 2) (30), CPC (version 0.9-r2) (31), and the intersection of both nonprotein-coding potential results were chosen as novel lncRNAs.

#### Identifying DE lncRNAs and Function Analysis

For each transcription region, the Fragment Per Kilobase of transcript per Million mapped reads (FPKM) value was calculated to quantify its expression abundance and variations, using RSEM software (32). The genes/transcripts with the parameter of corrected *P*-value (FDR) < 0.05 and |log_2_ (fold change)| > 1 were considered DE lncRNAs. The cis target genes of lncRNAs were predicted according to the location of gene transcripts (around 100 kb of lncRNA) in genomics. The correlation of expression between lncRNAs and protein-coding genes was analyzed to identify trans-target genes of lncRNAs and protein-coding genes with an absolute correlation over 0.999. In this study, DAVID (http://david.abcc.ncifcrf.gov/) online analysis software was used for GO function annotation and KEGG pathway enrichment analysis (33).

#### Construction of lncRNA-miRNA-mRNA ceRNA Network

Based on our previous Illumina HiSeq miRNA and mRNA sequencing data from the same samples, the lncRNA-miRNA-mRNA ceRNA regulatory network was constructed as per the following ceRNA theory: (a) expression correlation between mRNA and miRNA or lncRNA and miRNA was evaluated using the Spearman rank correlation coefficient (SCC). Pairs with SCC < −0.7 were selected as co-expressed negatively lncRNA-miRNA pairs or mRNA-miRNA pairs; (b) expression correlation between lncRNA and mRNA was evaluated using the Pearson correlation coefficient (PCC). Pairs with PCC > 0.9 were selected as co-expressed lncRNA-mRNA pairs; and (c) the hypergeometric cumulative distribution function test was used to test whether the common miRNA sponges between the two genes were significant. As a result, only the gene pairs with a *P* < 0.05 were selected. The ceRNA network related to muscle development was visualized using Cytoscape 3.7.1.

#### Real-Time Quantitative PCR Analysis

The total RNA was extracted from LT muscle samples of Tibetan sheep with TRIzol reagent (Invitrogen, Carlsbad, CA, USA), which was used for RNA-Seq and synthesized cDNA using a real-time quantitative PCR (RT-PCR) Kit (Takara, Dalian, China). RT-qPCR analysis was used to validate the authenticity transcriptome results. The primer sequences of genes were designed for RT-qPCR analysis (Table 1). The RNA samples that were the same as those used for the RNA-Seq, the RT-qPCR was conducted in triplicate using the SYBR Green Pro Taq HS qPCR Kit (Accurate Biology, Hunan, China) on an Applied Biosystems QuantStudio^®^ 6 Flex (Thermo Lifetech, MA, United States). The relative expression levels of these genes were analyzed using the 2^−ΔΔCt^ method. Sheep β-actin was used as an internal reference gene.

**Table 1:** A list of primers used for real-time quantitative PCR (RT-qPCR).

**lncRNAs**	**Forward (5' → 3')**	**Reverse (5' → 3')**	**Production length**
MSTRG.12273.7	AGCACCACAGGAACACGAC	GATGAGTATGCCCAGGATGA	100
MSTRG.11677.7	GCCAGCTCGTCCATCCTT	GCCTTTGTCCGTCGTTGC	112
MSTRG.12421.2	GCCCAAATCTTACCTTCCG	AAATGGCACGCCACTCCA	132
MSTRG.17123.1	CAGTTAGGTTTAGGTTTGGGTG	CCGGTCTCAGTCCGTTCTC	139
XR_003590204.1	GGGAGATGGAAGGAGGAG	TGCAGAATTAAGGTGGAGC	106
XR_003587855.1	TGCACTGTCTACGCTTTCC	AGGCATCCACTGGCTCCAC	131
XR_003589154.1	CATTCTTGGATACTTGGAGGTG	GAACGCTGGTTGGAGGAC	118
XR_003589363.1	CGCTCCATCATCCGAAAC	TCCAGCACCCAGTCACAAG	113
XR_003590685.1	TACTTCTCGCTGCCCTCG	ATGGTGGCTGGTGTCCTG	146
XR_003590346.1	AGAACAGAGGGCGTGGGA	AGGCTCGGCAGGATGAAG	102
β-actin	AGCCTTCCTTCCTGGGCATGGA	GGACAGCACCGTGTTGGCGTAGA	151

#### Correlation Analysis

To further verify the function of lncRNAs in fatty acid metabolism of the LT muscle in Tibetan sheep at different ages, Pearson correlation analysis was performed between 10 lncRNAs and fatty acids with significant differences. In this study, a two-tailed test was used for correlation analysis.

#### Statistical Analysis

All statistical analyses were performed using IBM SPSS 22.0 (SPSS, Inc., Chicago, IL, USA). The differences between the mean values were compared using the Duncan's multiple range test (*P* < 0.05). Each experiment was replicated at least three times.

## Results

### IMF and Fatty Acid Profile in Tibetan Sheep Meat at Different Ages

**Figure 1:**
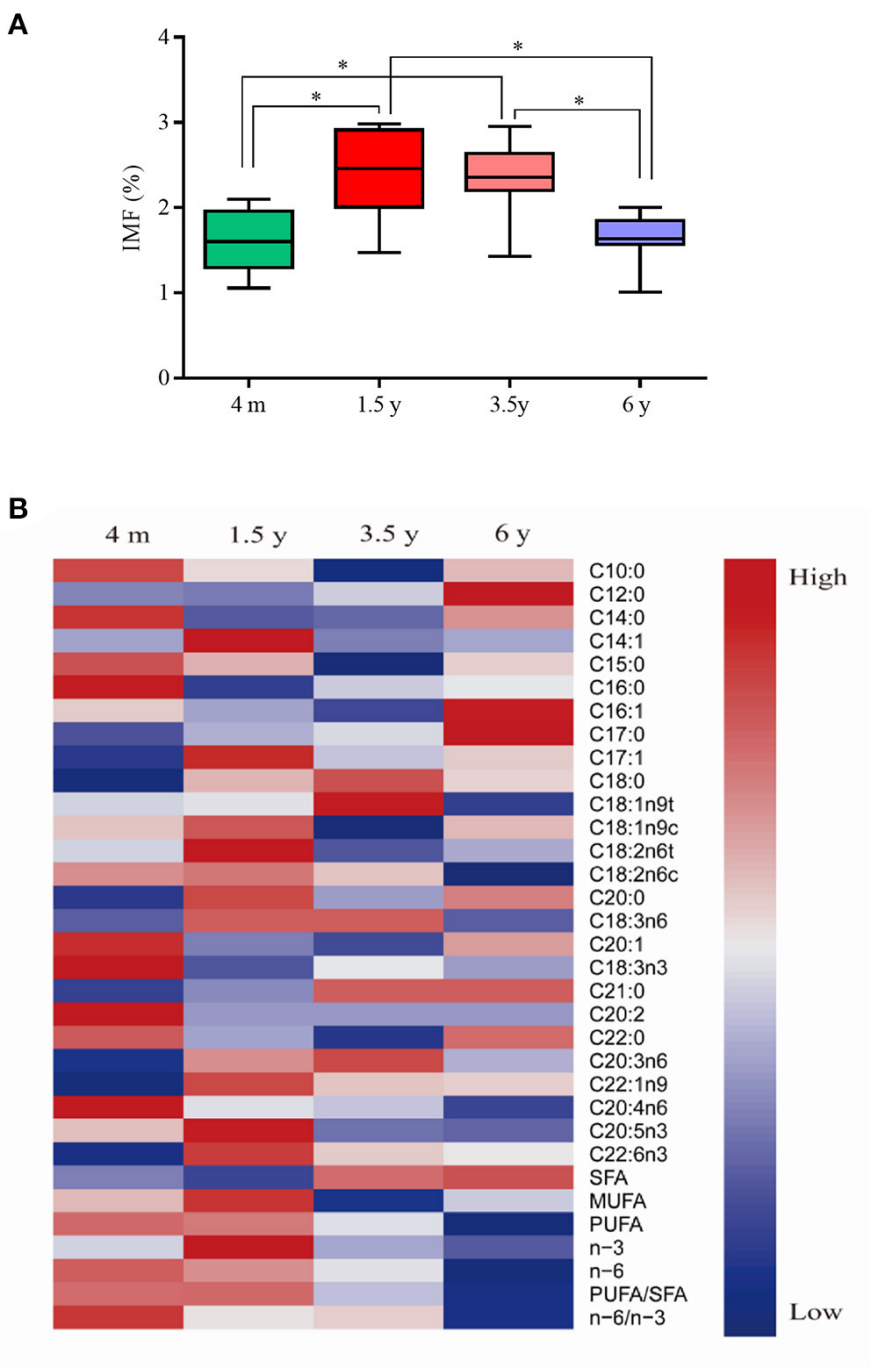
Intramuscular fat (IMF) and fatty acid content in Tibetan sheep meat at different ages. **(A)** Box plot of IMF content in Tibetan sheep meat at different ages. **(B)** Heat map of fatty acid composition in Tibetan sheep meat at different ages. *Difference is significant at *P* < 0.05.

Figure 1 shows the IMF and fatty acid content of Tibetan sheep at different ages. It seems that the IMF content in the LT muscles of Tibetan sheep increased first and then decreased with the growth of age (Figure 1A), it was higher of 1.5 y and 3.5 y than that of 4 m and 6 y (*P* < 0.05), and there was no significant difference between 1.5 y and 3.5 y (*P* > 0.05) and 4 m and 6 y (*P* > 0.05). A total of 26 fatty acids were detected in the LT muscle of Tibetan sheep at different ages (Figure 1B). The SFA content of 4 m and 1.5 y sheep was lower than the other two groups (*P* < 0.05), and there was no significant difference between 4 m and 1.5 y (*P* > 0.05), while SFA contents increased obviously at 3.5 y (*P* = 0.032). The content of monounsaturated fatty acid (MUFA), C14:1, C17:1, and C18:2n6t in Tibetan sheep meat of 1.5 y was significantly higher than that of other groups (*P* < 0.05), and it was also rich in polyunsaturated fatty acids.

### Quality Analysis of RNA-Seq Data

In this study, sixteen libraries were using the LT muscle tissues from four growth stages of Tibetan sheep. The summary statistics of the RNA-Seq data are shown in Table 2. The average of 91,167,071, 95,150,638, 90,313,673, and 87,472,429 raw reads were generated from four growth stages, respectively, after lower-quality reads were filtered, and more than 87 million clean reads were generated from each age. The correlation coefficient between four samples within 4 m, 1.5 y, 3.5 y, and 6.0 y of Tibetan sheep were 0.983, 0.890, 0.962, and 0.990, respectively. After removing the rRNA mapped reads, more than 86 million remaining clean reads were generated for each group. Of the remaining clean reads, more than an average of 82 million (92.04%) were mapped to the reference genome, and more than 74 million (91.75%) were unique mapped reads.

**Table 2:** The summary of the RNA-Seq data.

**Sample**	**Average raw reads**	**Average clean reads**	**Average remaining clean reads**	**Average mapped reads**	**Average unique reads**	**Average multiple reads**
4 m	91,167,071	90,738,055	90,373,003	88,176,017 (97.57%)	81,146,261 (92.03%)	7,029,756 (7.97%)
1.5 y	95,150,638	94,864,498	94,163,662	88,912,904 (94.42%)	81,647,895 (91.83%)	7,265,008 (8.17%)
3.5 y	90,313,673	89,970,024	89,518,448	82,392,489 (92.04%)	75,993,221 (92.23%)	63,99,268 (7.77%)
6 y	87,472,429	87,228,506	86,739,090	80,924,685 (93.30%)	74,245,776 (91.75%)	66,78,909 (8.25%)

**Figure 2:**
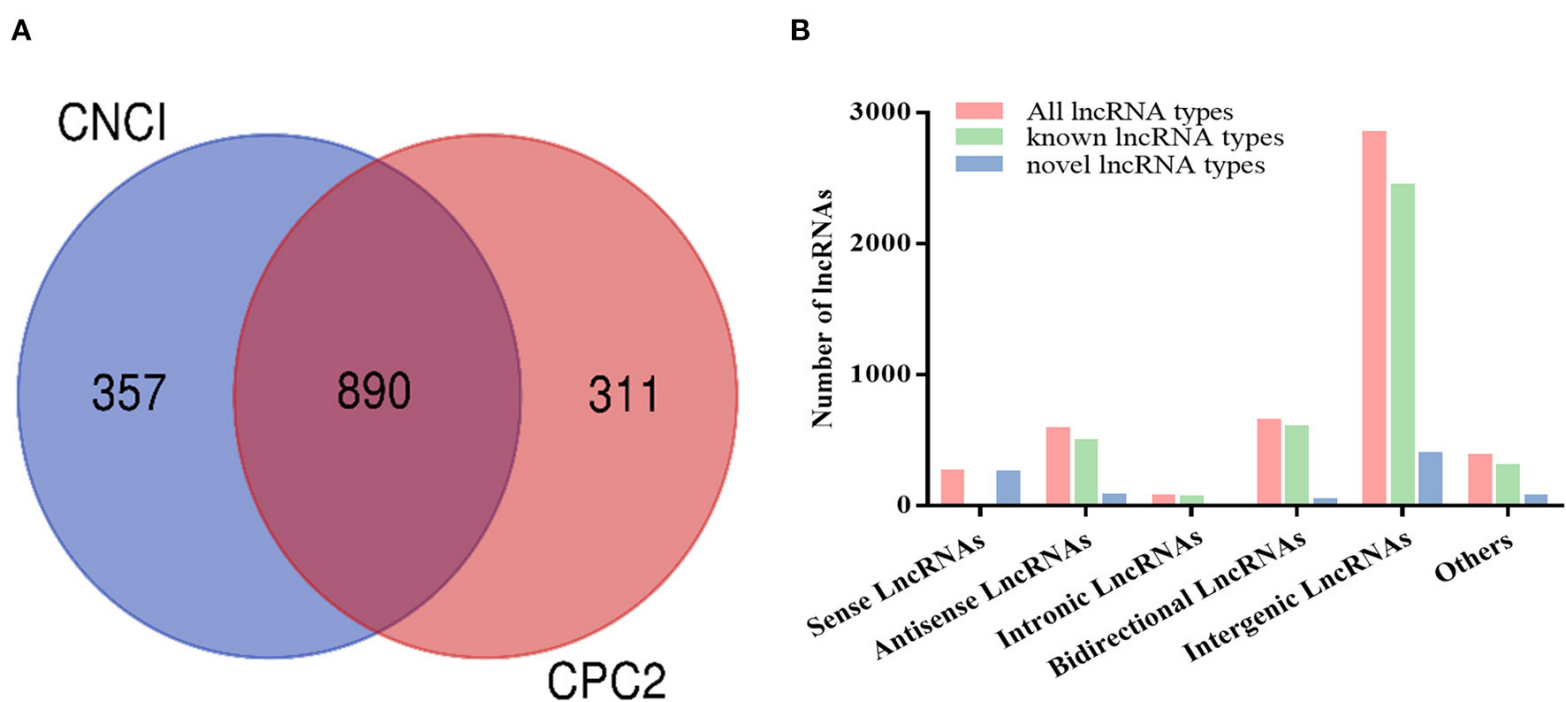
**(A)** Identification of 890 novel long noncoding RNAs (lncRNAs) without protein-coding potential evaluated by CNCI and CPC2. **(B)** LncRNA type distribution.

The mapping sequence of each library was reconstructed and assembled using StringTie software, and a total of 6,131 transcripts were obtained. The intersection of CPC2 and CNCI predicted that nonprotein-coding potential results were chosen as novel lncRNAs. A total of 890 novel lncRNAs were identified in this study (Figure 2A). According to the position of lncRNA in the genome relative to the protein-coding gene, lncRNAs were classified into five classes, namely, intergenic lncRNAs, bidirectional lncRNAs, intronic lncRNAs, antisense lncRNAs, and sense overlapping lncRNAs. The number of known lncRNAs and novel lncRNAs, i.e., 2,448 and 403, respectively, were the largest in the intergenic region (Figure 2B).

### Identification and Characteristics of lncRNAs During Intramuscular Lipid Metabolism in Tibetan Sheep

**Figure 3:**
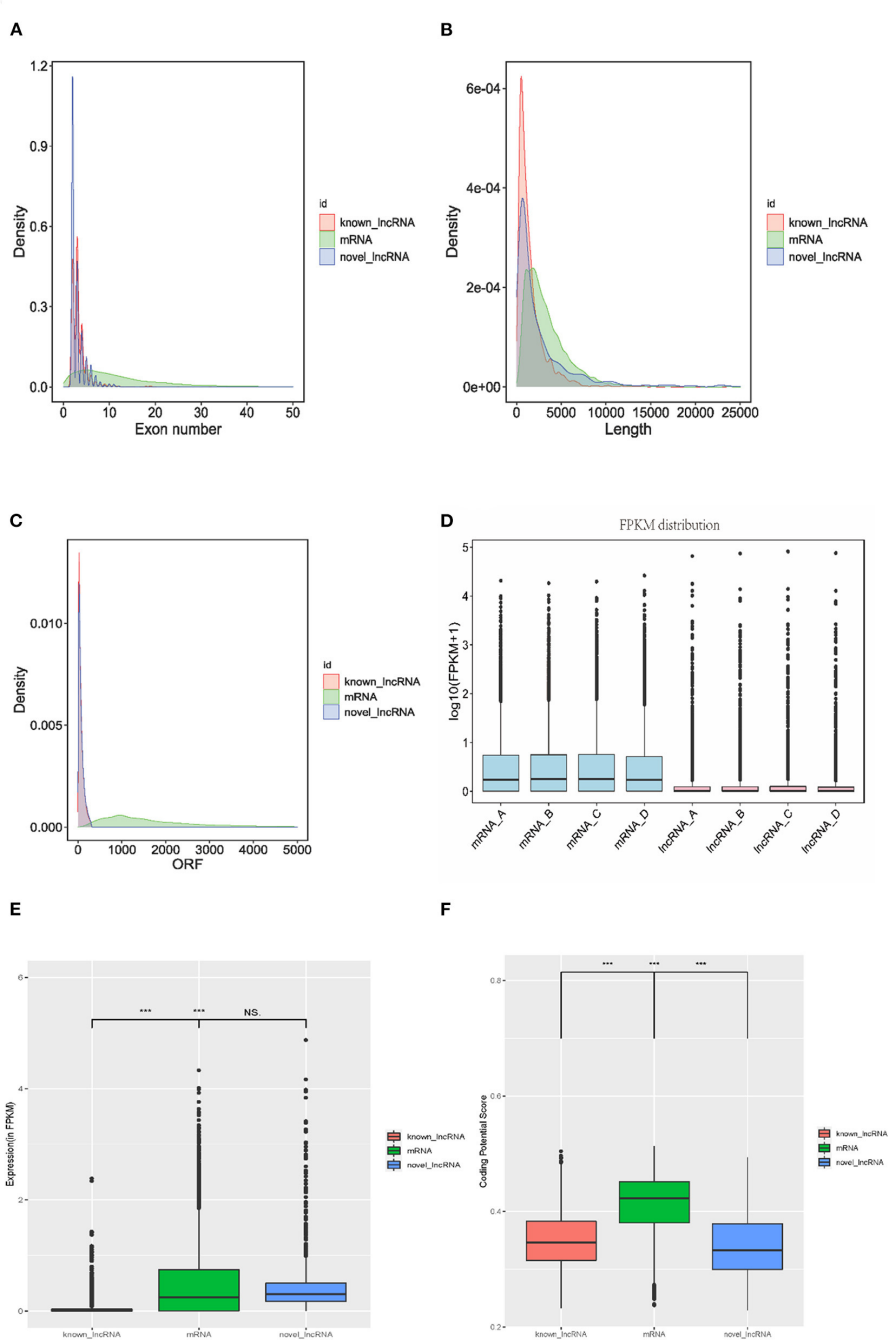
Characteristics of identified lncRNAs. **(A)** Distribution of exon numbers of mRNA, known lncRNAs, and novel lncRNAs. **(B)** Distribution of transcript lengths of mRNA, known lncRNAs, and novel lncRNAs. **(C)** Distribution of open reading frame (ORF) length of mRNA, known lncRNAs, and novel lncRNAs. **(D)** Box plot of log_10_ (FPKM+1) expression levels of lncRNA and mRNA in Tibetan sheep. mRNA_A, mRNA_B, mRNA_C, and mRNA_D was mRNA in 4-month-old, 4 m; 1.5-year-old, 1.5 y; 3.5-year-old, 3.5 y; and 6-year-old, 6 y of Tibetan sheep, respectively. lncRNA_A, lncRNA_B, lncRNA_C, and lncRNA_D were lncRNA in 4 m, 1.5 y, 3.5 y, and 6 y of Tibetan sheep, respectively. **(E)** Comparison of the expression levels of mRNA (green), known lncRNAs (red), and novel lncRNAs (blue), plotted as Fragments Per Kilobase of exon per Million fragments mapped (FPKM). ***P < 0.001; NS, no significant difference. **(F)** Coding potential scores of mRNAs, known lncRNAs, and novel lncRNAs calculated by coding potential calculator (CPC). ***P < 0.001.

To comprehensively explore the characteristics of lncRNAs during fat and fatty acid metabolism of Tibetan sheep meat, transcript length, the exon number, and open reading frame (ORF) length to explore differences of known and novel lncRNAs and mRNAs were characterized, as shown in Figures 3A–C. Similar to lncRNAs, the transcript length and ORF length of novel lncRNAs were significantly shorter than those of the mRNA transcript. In addition, the number of exons was also less than that of mRNA (*P* < 0.05). The expression level of lncRNA was much lower than that of mRNA (Figure 3D). The expression level (FPKM) of novel lncRNAs was higher than that of known lncRNAs (*P* < 0.001) but lower than that of mRNAs (*P* > 0.05) (Figure 3E). The novel lncRNAs possessed a coding potential score similar to that of known lncRNAs and a lower coding potential score than that of mRNAs (*P* < 0.001) (Figure 3F).

### DE lncRNAs During Intramuscular Lipid Metabolism in Tibetan Sheep

**Figure 4:**
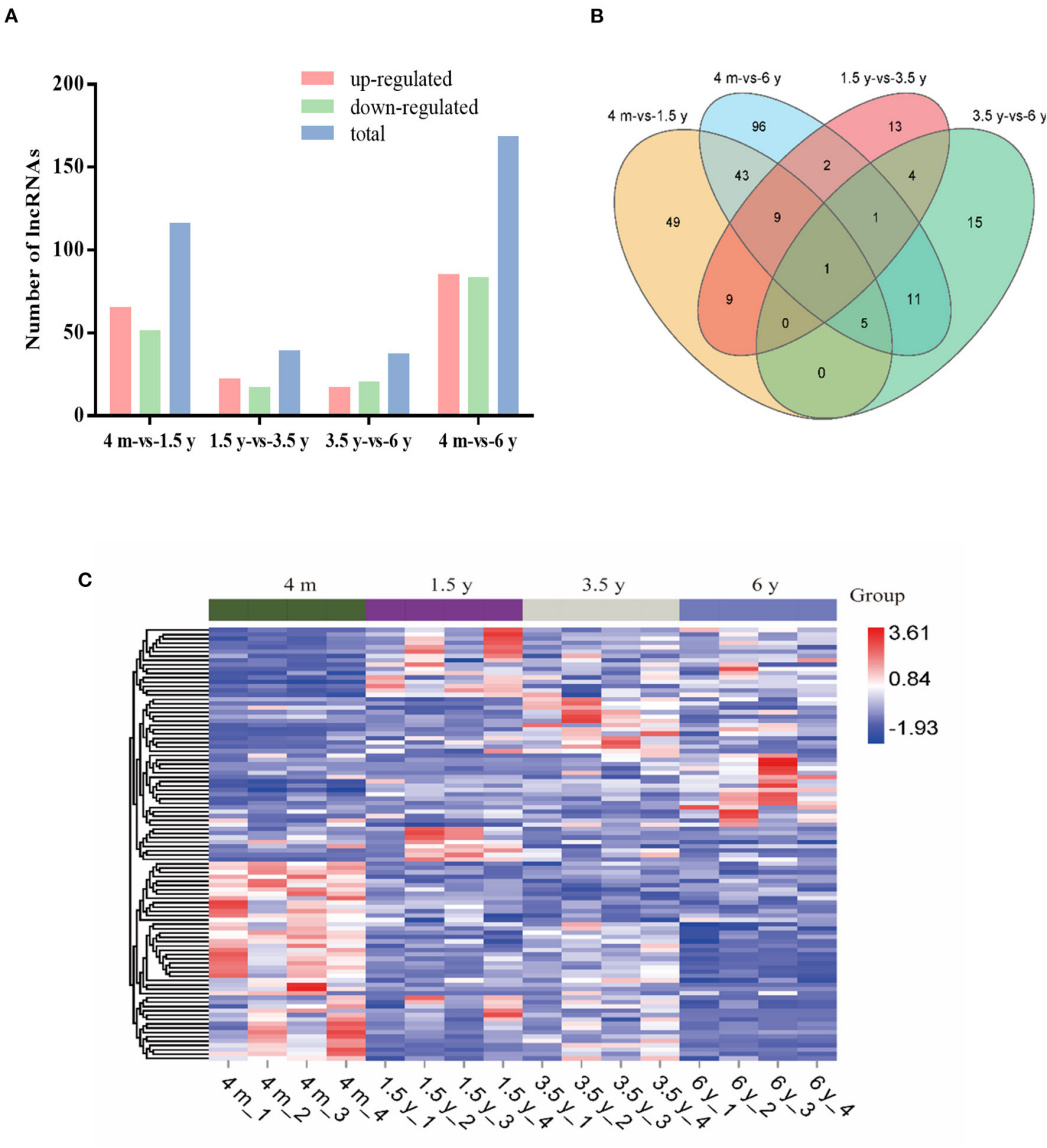
Analysis of the differentially expressed (DE) lncRNAs in the muscle of Tibetan sheep at different ages. **(A)** Statistical histogram of DE lncRNA in four contiguous period transcriptome comparative groups. **(B)** The Venn diagrams of the shared and unique differential lncRNAs in the four comparative groups. **(C)** Cluster heat map of DE lncRNA expression. Red means upregulation and blue means downregulation.

To find lncRNAs that were related to lipid metabolism, we compared the lncRNA expression levels of LT muscle of Tibetan sheep at different ages. Overall, 116 (65 upregulated; 51 downregulated), 39 (22 upregulated; 17 downregulated), 37 (17 upregulated; 20 downregulated), and 168 (85 upregulated; 83 downregulated) DE lncRNAs were identified in 4 m vs. 1.5 y, 1.5 y vs. 3.5 y, 3.5 y vs. 6 y, and 4 m vs. 6 y groups, respectively (Figure 4A). There was a lncRNA co-expressed in four contiguous period transcriptome comparative groups (Figure 4B). In addition, the heat map results of DE lncRNAs showed that there were obvious differences in lncRNA among the four ages (Figure 4C). The results demonstrated that the differences in lipid metabolism in the muscles of Tibetan sheep at different ages might be caused by these DE lncRNAs.

### Functional Characterization of DE lncRNAs During Intramuscular Lipid Metabolism in Tibetan Sheep

**Figure 5:**
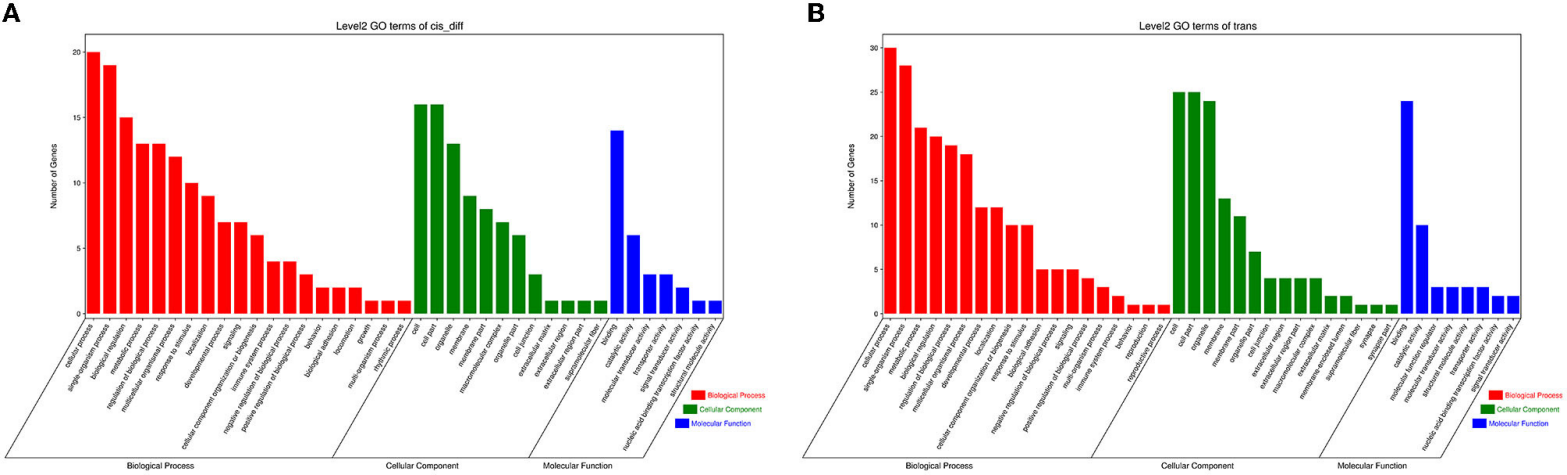
Histogram of DE lncRNA target genes of gene ontology (GO) functional annotation. The GO annotation included biological process (BP), molecular function (MF), and cellular component (CC). **(A)** The target genes in the *cis*-mRNA pairs. **(B)** The target genes in the *trans*-mRNA pairs.

To systematically study the functions of lncRNA and fatty acid metabolism in LT muscle of Tibetan sheep, the target genes of DE lncRNA were analyzed. In total, 39 significantly different *cis*-mRNA pairs and 62 significantly different *trans*-mRNA pairs were identified. To further analyze the potential function of DE lncRNA in regulating lipid metabolism in Tibetan sheep LT muscle, GO function annotation and KEGG enrichment analysis were performed on *cis*-regulated and trans-regulated target genes, respectively. The results indicated that most of the target genes in the *cis*-mRNA pairs were significantly enriched into specific functional groups (*P* < 0.05), including those that were related to muscle development, external stimuli, and immune (Figure 5A). GO term involved in the function of muscle structure development (GO:0061061) and antibacterial peptide production (GO:0002778). Most of the target genes in the *trans*-mRNA pairs were significantly enriched into protein modification and defense functions (*P* < 0.05) (Figure 5B). GO term involved in the function of protein palmitoylation (GO:0018345) and defense response to fungus (GO:0050832). These enriched functions were all related to muscle development.

The KEGG pathway was used to further analyze the potential functional signaling pathways of target genes in the four compare groups of the LT muscle of Tibetan sheep (Table 3), which is a substitute method of gene function classification focusing on biochemical pathways. The results showed that most of the target genes in the *cis*-mRNA pairs were significantly enriched in the immune system, development, and signal transduction (*P* < 0.05). However, most of the target genes in the *trans*-mRNA pairs were significantly enriched in the protein digestion and absorption and fatty acid biosynthesis (*P* < 0.05). *FASN* was significantly enriched in fatty acid biosynthesis (*P* < 0.05). The results demonstrated that DE lncRNA *trans*-regulate target genes played an important biological function in the LT muscle growth and development and intramuscular fatty acid metabolism of Tibetan sheep at different ages.

**Table 3:** Significant results of KEGG enrichment analysis.

**KO**	**Description**	**Pathway**	***P*-value**	**Q-value**	**Involved DEGs**
*cis*-mRNA
ko04713	Environmental adaptation	Circadian entrainment	0.01505808	0.3560576	PER3; FOS
ko04625	Immune system	C-type lectin receptor signaling pathway	0.02070466	0.3560576	CYLD; EGR2
ko04660	Immune system	T cell receptor signaling pathway	0.02104089	0.3560576	PAK4; FOS
ko04668	Signal transduction	TNF signaling pathway	0.02561897	0.3560576	FOS; NOD2
ko04380	Development	Osteoclast differentiation	0.02939399	0.3560576	CYLD; FOS
ko04915	Endocrine system	Estrogen signaling pathway	0.02939399	0.3560576	HSPA1B; FOS
*trans*-mRNA
ko04974	Digestive system	Protein digestion and absorption	0.006172466	0.4073828	COL13A1; COL11A1; COL22A1
ko04711	Environmental adaptation	Circadian rhythm-fly	0.01630492	0.4414381	PER2
ko00061	Lipid metabolism	Fatty acid biosynthesis	0.03764888	0.4414381	FASN

### Construction of lncRNA-miRNA-mRNA Regulatory Network

**Figure 6:**
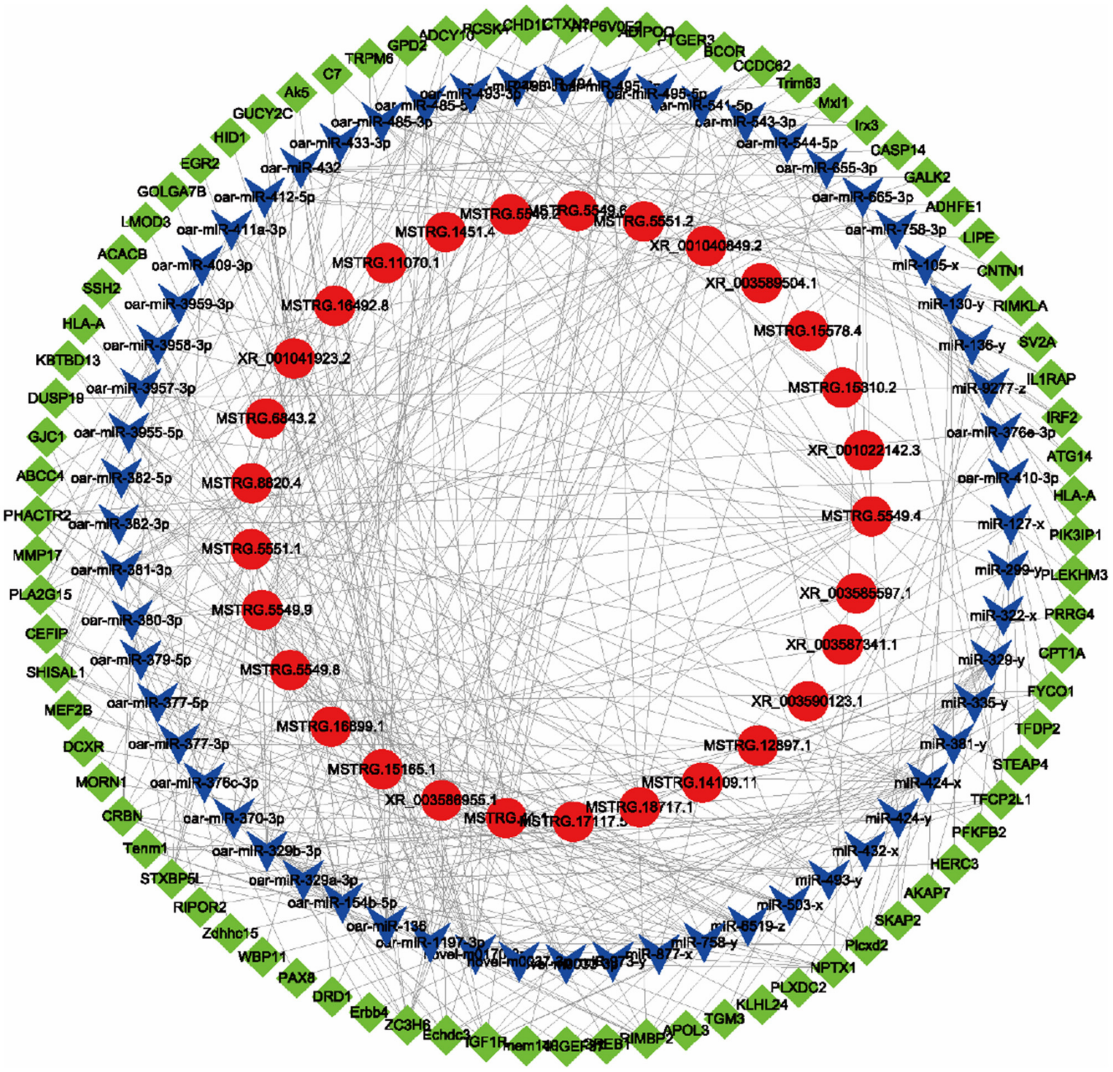
The lncRNA-miRNA-mRNA ceRNA regulatory network related to fat and fatty acid metabolism in Tibetan sheep muscle. The red circle represents lncRNAs. Blue V represents miRNAs. Green diamonds represent mRNAs.

To further analyze the biological functions of these DE lncRNAs, based on the ceRNA hypothesis mechanism, by integrating the results of our previous miRNA and mRNA sequencing data, a regulatory network of lncRNA-miRNA-mRNA was constructed. The ceRNA network contained 166 lncRNA-miRNA pairs and 250 miRNA-mRNA pairs and included 29 lncRNAs, 53 miRNAs, and 81 mRNAs (Figure 6). Among them, the ncbi_443090 (*GPD2*), ncbi_100169699 (lipase E hormone-sensitive enzyme (*LIPE*)), ncbi_101105870 (*TFDP2*), ncbi_443434 (*CPT1A*), ncbi_101114816 (*ACACB*), and ncbi_101111848 (*ADIPOQ*) and other mRNAs related to fatty acid and lipid metabolism were identified. Finally, some important regulatory relationships related to fatty acid metabolism were obtained through connectivity and correlation: MSTRG.41.1-miR-381-3p-*GPD2*, XR_001040849.2-miR-136-*GPD2*, XR_003590307.1-miR-485-3p-*TFDP2*, XR_003587341.1-miR-127-5p-*TFDP2*, MSTRG.1451.4-novel-m0170-3p-*ACACB*, XR_003585597.1-miR-376c-3p-*ADIPOQ*, MSTRG.5551.2-miR-105-5p-*CPT1A*, and XR_001041923.2-miR-412-5p-*LIPE*. We speculated that these lncRNAs regulated the corresponding miRNAs and further regulated genes related to fatty acids and IMF, which played an important biological role in fatty acid and fat metabolism in Tibetan sheep muscle.

### GO Function Annotation and KEGG Enrichment Analysis of mRNA in ceRNA Network

**Figure 7:**
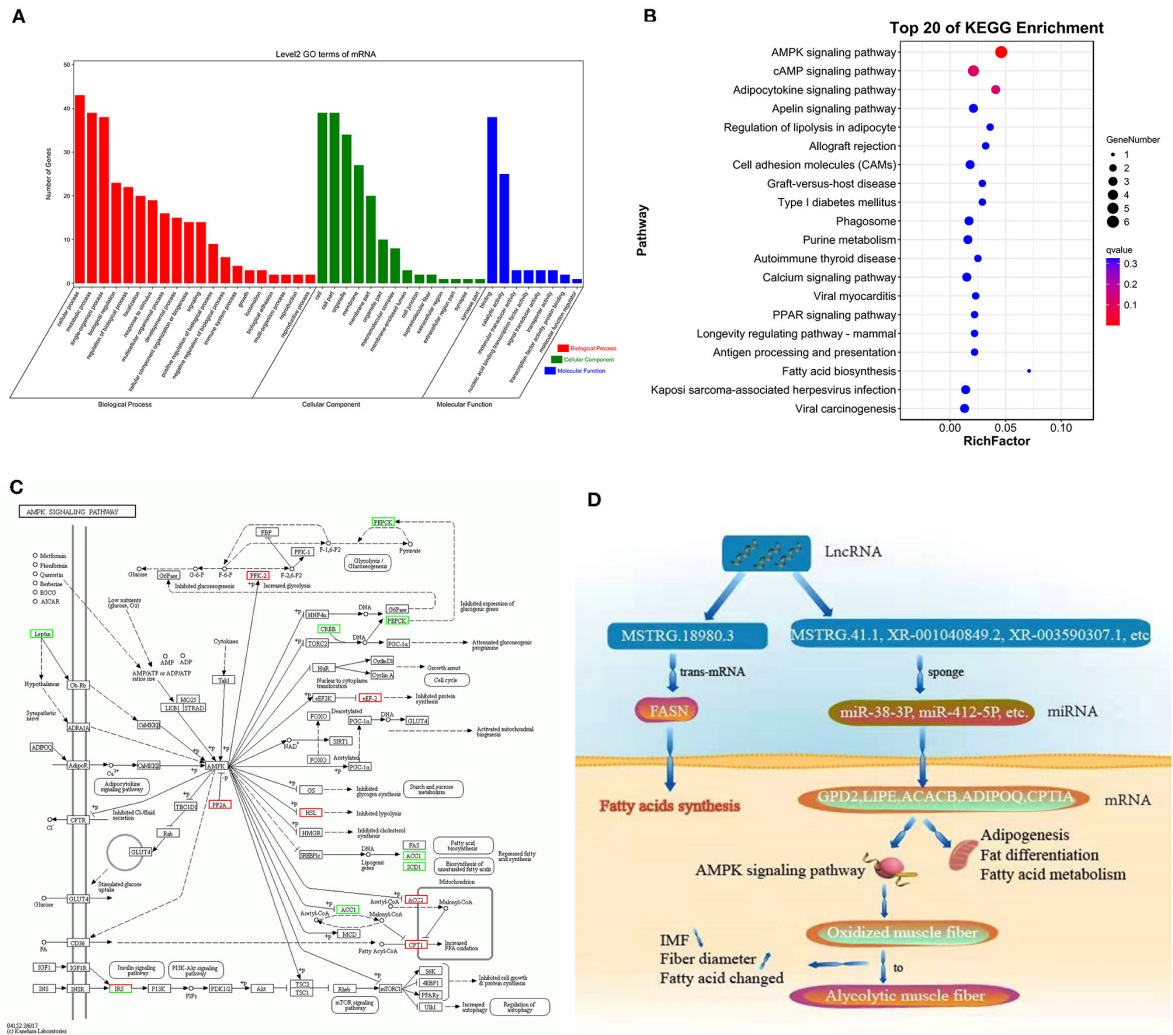
**(A)** Histogram of mRNA GO function annotated in ceRNA network. **(B)** Bubble chart of Kyoto encyclopedia of genes and genomes (KEGG) enrichment analysis. **(C)** AMPK signaling pathway during the growth and development of Tibetan sheep. **(D)** Model of lncRNA regulating lipid metabolism in Tibetan sheep.

To further analyze the potential function of mRNA in the ceRNA network in fatty acid and fat metabolism in Tibetan sheep muscle, we performed GO function annotation and KEGG enrichment analysis with 81 mRNAs in the ceRNA network. The results showed that most of the genes were enriched into specific functional groups, mainly including processes such as metabolism and protein modification (Figure 7A). GO term involved in the function of lipid metabolic process (GO:0019216), where *ADIPOQ*, and *ACACB*, and lipid metabolism-related genes were significantly enriched in this pathway (*P* < 0.05). In addition, it was also significantly enriched in the regulation of fatty acid metabolic process (GO:0019217) and cellular lipid metabolic process (GO:0044255) (*P* < 0.05). These functions were all related to lipid metabolism. The genes in these GO functions might further regulate fatty acid metabolism during the growth and development of Tibetan sheep. The results of KEGG enrichment analysis showed that the most significant enrichment was the AMPK signaling pathway (*P* = 0.0000112361) (Figure 7B), and *LIPE, ADIPOQ, ACACB*, and *CPT1A* were enriched in the AMPK signaling pathway (*P* < 0.05). Additionally, our previous study found that the AMPK signaling pathway was related to the transformation of muscle fiber types in LT muscles of Tibetan sheep (Figure 7C). Therefore, we speculated that these genes might be involved in the AMPK signaling pathway, changed the types of muscle fibers in Tibetan sheep muscles, and ultimately lead to differences in fatty acid composition and IMF content in LT muscles of Tibetan sheep (Figure 7D). In addition, the signaling pathways that were significantly enriched include the adipocytokine signaling pathway, PPAR signaling pathway, and fatty acid biosynthesis signaling pathway. These signaling pathways were related to lipid metabolism and further regulate fatty acid metabolism in LT muscle of Tibetan sheep at different ages.

### Validation of lncRNA Expression by RT-qPCR

**Figure 8:**
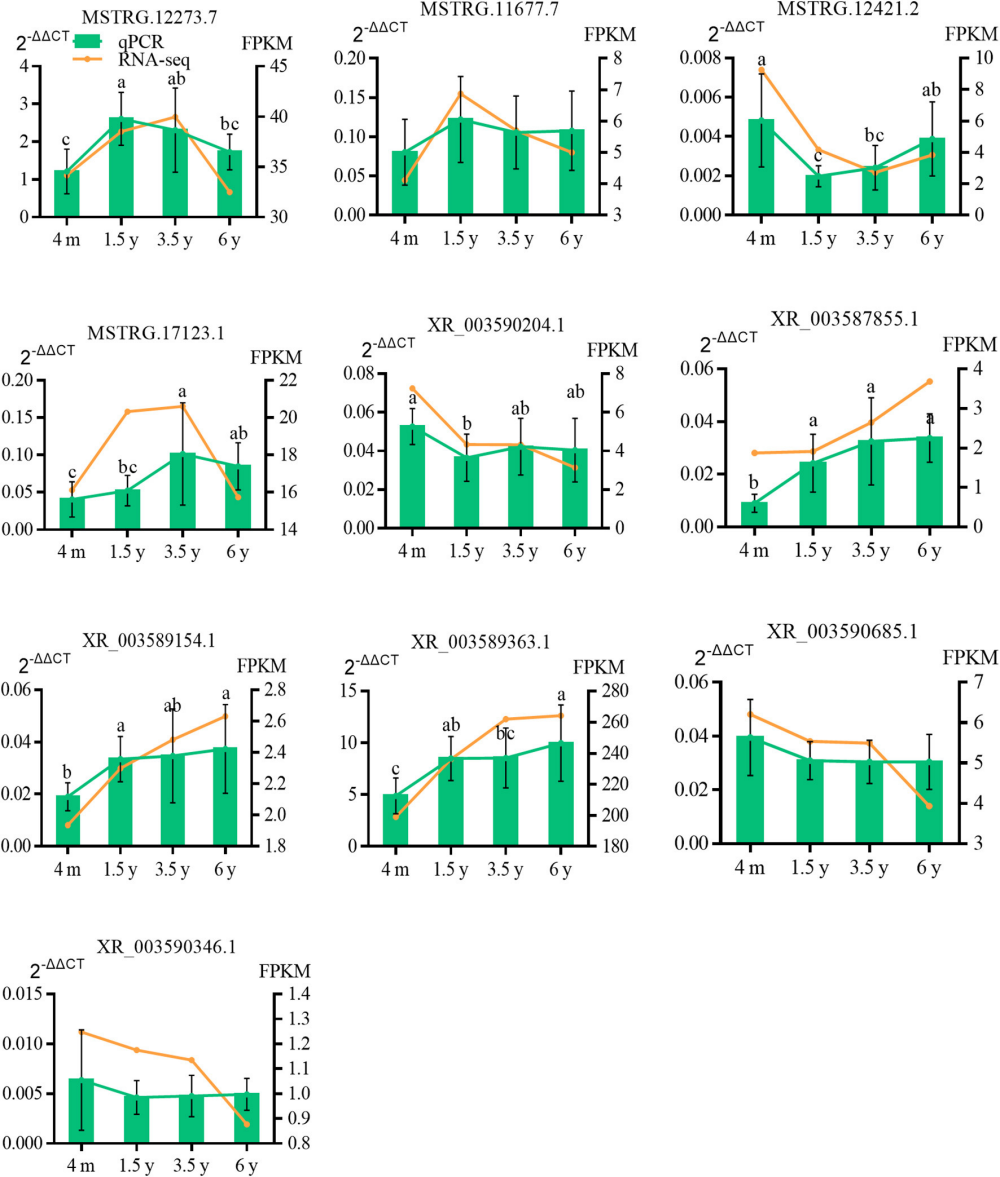
Comparison of the expression levels of lncRNA between RNA-Seq and RT-qPCR. RT-qPCR data were shown as the means ± S.D. Different letters (a–c) indicate significant difference between different ages (*P* < 0.05). 2^−ΔΔCT^ was the result of RT-qPCR, and FPKM was the result of RNA-Seq.

To verify the RNA-Seq results, 10 DE lncRNAs were randomly selected and verified by RT-qPCR (Figure 8). The RT-qPCR expression pattern of selected genes was consistent with the results of RNA-Seq analysis, and this demonstrated the reliability and accuracy of the RNA-Seq method used in this study.

### The Relationship Between lncRNAs and Fatty Acids

**Figure 9:**
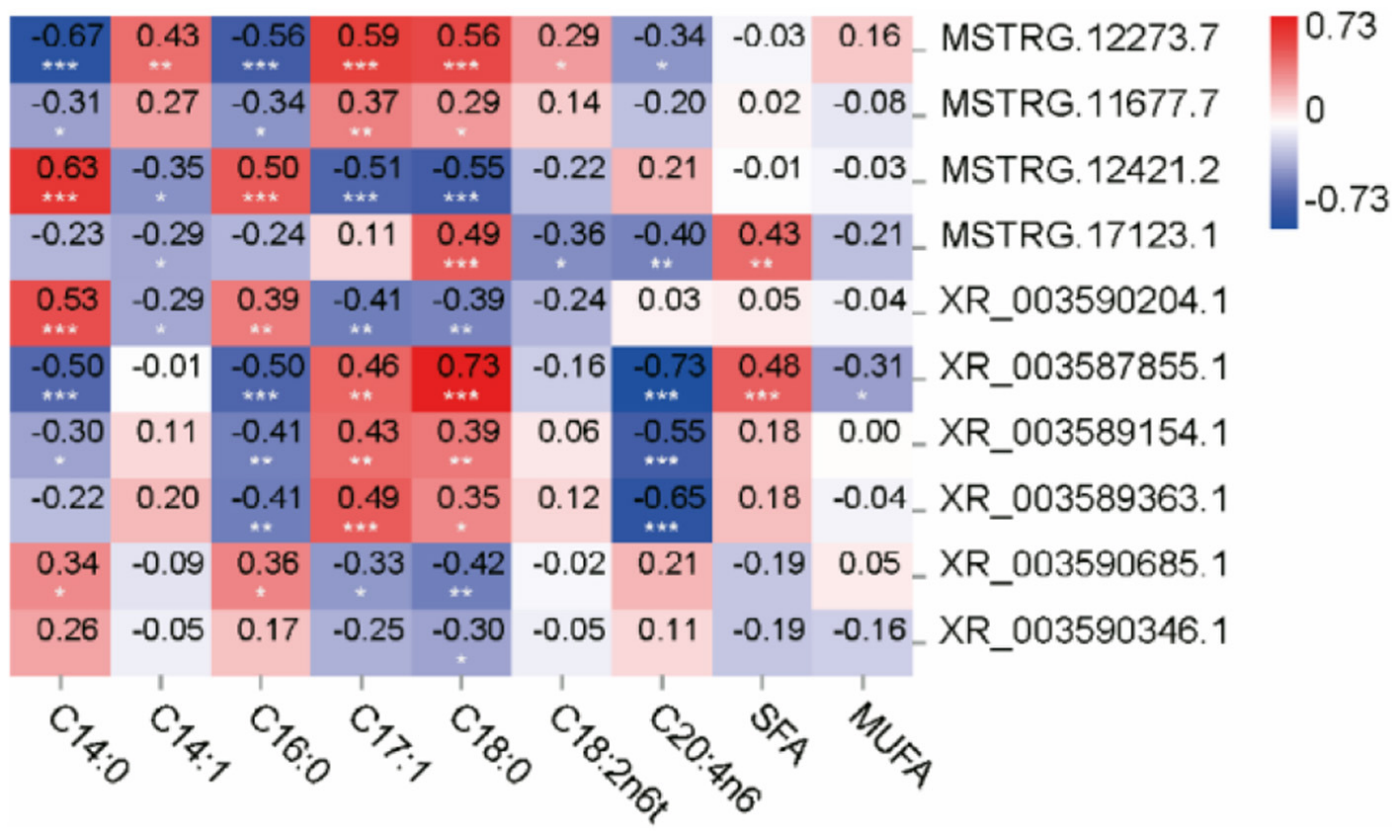
Pearson correlations between expression of lncRNAs and content of fatty acids in the muscle of Tibetan sheep. *Correlation is significant at *P* < 0.05, **correlation is significant at *P* < 0.01, and ***correlation is significant at *P* < 0.001.

In this study, fatty acids with significant differences were selected, and correlation analysis was performed with the 10 lncRNAs. It was found that there was a very significant correlation between most fatty acids and lncRNAs (Figure 9). Positive correlation was found between the C14:0 and MSTRG.12421.2 (0.63, *P* < 0.001), XR_003590204.1 (0.53, *P* < 0.001), while negative correlation was found between C14:0 and MSTRG.12273.7 (−0.67, *P* < 0.001), XR_003587855.1 (−0.50, *P* < 0.001). It was found between C16:0 and MSTRG.12421.2 (0.50, *P* < 0.001), while it was found between C16:0 and MSTRG.12273.7 (−0.56, *P* < 0.001). A positive correlation was found between C17:1 and MSTRG.12273.7 (0.59, *P* < 0.001); however, a negative correlation was found between C17:1 and MSTRG.12421.2 (−0.51, *P* < 0.001). A positive correlation was found between C18:0 and XR_003587855.1 (0.73, *P* < 0.001), and a negative correlation was also found between C20:4n6 and XR_003587855.1 (−0.73, *P* < 0.001).

## Discussion

Lipid metabolism in animal skeletal muscle has a significant impact on metabolic energy and homeostasis. In addition, TMF and fatty acid profiles have positive impacts on flavor, juiciness, tenderness, and nutrients. Skeletal muscle accounts for 40–50% of animal body weight, and the skeletal muscle development process of mammal prenatal had been studied more clearly (34, 35). The skeletal muscle development of animal postnatal mainly involved an increase in muscle fiber diameter and transformation of muscle fiber types (36, 37). Additionally, oxidized muscle fibers gradually transformed into glycolytic muscle fibers with the increase of age. In this study, the IMF content of Tibetan sheep increased first and then decreased with the increase of age. In addition, it was largest at 1.5 y, which might be related to the transformation of oxidized muscle fibers into glycolytic muscle fibers. In addition, higher MUFA and PUFA contents were observed in 1.5 y of Tibetan sheep. The above results demonstrated that 1.5 y was a more suitable slaughter age of Tibetan sheep for a healthy human diet.

Fatty acids are important nutrients for human health and have important physiological functions. MUFA can reduce total cholesterol, enhance antioxidant enzyme activity, and reduce blood pressure and blood sugar level, preventing memory loss and promoting growth and development. It is of great significance for reducing cardiovascular diseases (38). More studies have demonstrated that lncRNA has a significant effect on lipid metabolism. Analysis of the functional role of lncRNA in the regulation of lipid and fatty acid metabolism is helpful to explore the mechanisms of lipid deposition and adipose tissue development.

In this study, we systematically analyzed the lncRNA profile related to fat and fatty acid metabolism in the LT muscles of Tibetan sheep at different growth and developmental stages. A total of 890 novel lncRNAs were identified, with similar characteristics to that of 3,943 known lncRNAs, in order to further analyze the molecular mechanism that caused the differences in IMF and fatty acids profile in the LT muscle growth and development of Tibetan sheep. We analyzed the DE lncRNAs in the four contiguous period transcriptome comparative groups, and a total of 360 DE lncRNAs were identified. KEGG enrichment analysis results found that DE lncRNA *trans*-regulated the target genes to regulate LT muscle development and fatty acid biosynthesis. In addition, *COL13A1, COL11A1, COL22A1*, and *FASN* were significantly enriched in muscle development-related signaling pathways and fatty acid biosynthesis pathway. Among them, *COL13A1, COL11A1*, and *COL22A1* are important members of the collagen superfamily. Previous studies found that the mutation of *COL13A1* during muscle development might cause myasthenia syndrome (39). Nallanthighal et al. (40) found that *COL11A1* upregulated fatty acid β oxidation in ovarian cancer cells. In addition, *COL11A1* had been demonstrated to enhance the expression of proteins involved in fatty acid synthesis and induce upregulation of fatty acid biosynthesis (40). Charvet et al. (41) found that knocking out *COL22A1* caused zebrafish muscle weakness and contraction-induced fiber detachment (41). Moreover, *FASN* had also been significantly enriched in fatty acid biosynthesis signaling pathways, which are a fatty acid synthase and a key rate-limiting enzyme in the fatty acid synthesis process *de novo* (42). These studies confirmed that MSTRG.18980.3, XR_003585779.1 (LOC105606646), MSTRG.11734.2, and MSTRG.18980.3 *trans*-regulated *COL13A1, COL11A1, COL22A1*, and *FASN*, respectively, and were involved in LT muscle growth and development and fatty acid metabolism, which played an important biological function.

Previous studies have demonstrated that lncRNA could serve as a miRNA sponge to indirectly regulate the expression of downstream target genes of miRNA (43). Song et al. (44) found that lncRNA-KRTAP5-AS1 and lncRNA-TUBB2A could serve as competing endogenous RNA to influence the function of *Claudin-4* (44). Therefore, we constructed the lncRNA-miRNA-mRNA ceRNA regulatory network to reveal the functions of these DE lncRNAs in IMF and fatty acid metabolism in Tibetan sheep muscle at different growth and development stages. A total of 81 mRNAs were identified in the ceRNA network. Among them, *GPD2* could catalyze the esterification of fatty acids to triglycerides (45). In addition, *GPD2* was potentially regulated by the MSTRG.41.1-miR-381-3p and XR_001040849.2-miR-136 pairs. The study about miR-381-3p mainly focuses on diseases. Previous studies suggested that miR-381-3p was a dual inhibitor of apoptosis and necrosis, and it could also regulate downstream target genes to inhibit smooth muscle growth of human (46). However, miR-136 may be related to fat metabolism, one of the evidence is that overexpression of miR-136 could inhibit the expression of LRH-1 and lead to dyslipidemia and liver steatosis (47). The results of this study showed that MSTRG.41.1-miR and XR_001040849.2 regulated miR-381-3p and miR-136, respectively, relieved the inhibition of *GPD2* by miRNA, and played an important biological role in fat metabolism. *TFDP2* blocked the differentiation of adipocytes by inhibiting the binding of *CEBPA* to the promoters of target genes (48). *TFDP2* was potentially regulated by the relationship pair of XR_003590307.1-miR-485-3p and XR_003587341.1-miR-127-5p in this study. There were many studies on miR-485-3p in diseases. Previous studies showed that miR-485-3p was involved in the regulation, apoptosis, and migration of metastatic tumors. Overexpression of miR-485-3p in fibroblasts could promote fibrosis (49). Ji et al. (50) demonstrated that miR-127-5p plays an important regulatory role in fat deposition by regulating downstream target genes (50). *CPT1A* is a key enzyme for carnitine-dependent transport across the inner mitochondrial membrane. Lack of *CPT1A* would reduce the rate of fatty acid oxidation (51). MSTRG.5551.2 acted as a miR-105-5p sponge to further regulate *CPT1A* in this study. miR-105-5p played an important role in regulating the proliferation, migration, and apoptosis of cancer cells (52). *ACACB* is acetyl-CoA carboxylase β and played an important role in fatty acid metabolism (53). *ACACB* was potentially regulated by the MSTRG.1451.4-novel-m0170-3p relationship pair in this study. The novel-m0170-3p was a novel miRNA identified, and there was no study on novel-m0170-3p. *ADIPOQ* is related to lipid metabolism and adipocyte differentiation (54, 55). *ADIPOQ* was potentially regulated by the XR_003585597.1-miR-376c-3p relationship pair in this study. Zhang et al. (56) found that LncRNA-LINC00152 was downregulated by miR-376c-3p to further limit the viability of rectal cancer cells and promote cell apoptosis (56). In addition, *LIPE* is a kind of lipid decomposing enzyme, which plays a key role in regulating the deposition of adipose tissue (57). The *LIPE* gene was potentially regulated by the XR_001041923.2-miR-412-5p relationship pair in this study. There were few studies on the lipid metabolism of miR-412-5p; however, previous studies suggested that the inactivation of miR-412-5p in vascular endothelial cells lead to the high expression of Xpo1 and the inhibition of the p53-p66SHC-p16 pathway, which ultimately promoted the formation of vascular endothelial cells and hemorrhoid blood vessels (58). These genes were involved in fatty acid and lipid metabolism during the growth and development of Tibetan sheep.

Long noncoding RNA acts as a sponge of miRNA and played an important role in the process of lipid metabolism. The enrichment analysis of mRNA in the ceRNA regulatory network found that the AMPK signaling pathway was most significantly enriched. There have been numerous reports on the function of the AMPK signaling pathway. van der Vaart et al. (59) found that AMPK signaling played an important role in brown adipose tissue activation (59). Dan et al. (60) found AMPK signaling and inhibiting complex I in the mitochondria, leading to a reduction in mitochondrial respiration and elevated ATP production (60). In addition, the latest studies found that the AMPK signaling pathway played a key role in the regulation of skeletal muscle fiber type transformation (61, 62). Therefore, combined with our previous study, we speculated that the oxidized muscle fibers gradually transformed into glycolytic muscle fibers in Tibetan sheep muscle were the direct cause of poor meat quality. Moreover, it was significantly enriched into adipocytokine signaling pathway, PPAR signaling pathway, and fatty acid biosynthesis signal pathway.

The function of lncRNA in fatty acid metabolism was estimated through the correlation analysis between lncRNA and fatty acid. Negative correlations were found between XR_003589363.1 (RPS28) and C20:4n6 (*r* = −0.65, *P* < 0.001), and positive correlations were found between XR_003589363.1 (RPS28) and C17:1 (*r* = 0.49, *P* < 0.001). *RPS28* is a kind of Ribosomal proteins that regulate protein biosynthesis and may be associated with muscle development. Jiao et al. (63) found that Rps28a is regulating the levels of a subset of proteins with known antiaging roles in skeletal muscle, while there was less study on fatty acid metabolism (63). The above studies demonstrated that lncRNA played a direct or indirect biological role in the metabolism of lipids and fatty acids in the LT muscles of Tibetan sheep during growth and development. However, these potential regulatory mechanisms still need to be further studied.

## Conclusion

In summary, the results of this study indicated that the higher MUFA and PUFA content were observed in 1.5 y of Tibetan sheep, which demonstrated that 1.5 y was a more suitable slaughter age of Tibetan sheep for a healthy human diet. Furthermore, the reasons for this difference through the deep sequencing and bioinformatic analysis of the skeletal muscles of Tibetan sheep at different growth and development stages were analyzed. The results showed that DE lncRNA *trans*-regulated *FASN*, to regulate fatty acid metabolism during the growth and development of Tibetan sheep. Moreover, lncRNA also acted as a miRNA sponge, regulated *GPD2, TFDP2, LIPE, CPT1A, ACACB*, and *ADIPOQ*, and played an important biological role in lipid and fatty acid metabolism. Among them, *LIPE, ADIPOQ, ACACB*, and *CPT1A* changed the formation of energy metabolism in skeletal muscle through the AMPK signaling pathway, and oxidized muscle fibers were gradually transformed into glycolytic muscle fibers, which caused an increase in Tibetan sheep muscle fiber diameter and reduction in the IMF content, meat tenderness, and meat juiciness. In addition, it also changed the metabolism of fatty acid composition, resulting in differences in fatty acid profiles in Tibetan sheep muscles at different growth stages.

## Data Availability Statement

The datasets presented in this study can be found in online repositories. The names of the repository/repositories and accession number(s) can be found in the article/supplementary material.

## Ethics Statement

The animal study was reviewed and approved by the Faculty Animal Policy and Welfare Committee of Gansu Agricultural University (Ethic approval file No. GSAU-Eth-AST-2021-001).

## Author Contributions

GB did the data analysis and wrote the manuscript. SL and FZ performed the investigation and collected the samples. JW, XL, JH, BS, YW, and LZ performed the formal analysis, methodology, and software. YL and SL did the project administration and revised the manuscript. All authors contributed to this study and approved the submitted version.

## Funding

This study was supported by the fund of Distinguished Young Scholars Fund of Gansu Province (21JR7RA857), the Fuxi Young Talents Fund of Gansu Agricultural University (Gaufx-03Y04), the Projects of Gansu Agricultural University (GSAU-ZL-2015-033), and the Key R&D Projects in Gansu Province (18YF1WA082).

## Conflict of Interest

The authors declare that the research was conducted in the absence of any commercial or financial relationships that could be construed as a potential conflict of interest.

## Publisher's Note

All claims expressed in this article are solely those of the authors and do not necessarily represent those of their affiliated organizations, or those of the publisher, the editors and the reviewers. Any product that may be evaluated in this article, or claim that may be made by its manufacturer, is not guaranteed or endorsed by the publisher.
